# Supplementary material for: Chromothripsis during telomere crisis is independent of NHEJ, and consistent with a replicative origin
Source: Genome Res. 2019 May;29(5):737–49. doi: 10.1101/gr.240705.118 (PMC6499312; doi:10.1101/gr.240705.118)
Supplement: Supplemental Material [file supp_gr.240705.118_Supplemental_file_1.zip › contigs/annotated_contigs/DB99/contig.2.DB99_length_274_mean_cov_1.64233576642.docx]

**DB99_length_274_mean_cov_1.64233576642**

```
TTTCTTTCTTCATTTCACCGTTGTATCTCCAGGTCTTTGATCCACTCTAAGAATGGCTTCTCAGCTCTGTTCCTTGT|CACCACCAC|C
 >chr10:127890256-127890342 - E=2e-40 p=4e-02                                 >chr10:12
CTGCCCCCATCACGTACACAAAATCTAAGTCCTAACAACTCTCCAACGTCCATCTCAAACCAAATCCTGGGCTCTTTCTGTAGAGTCTT
7889271-127889446 - E=4e-73
GGACTCTCCAACCTATTGAGAAGCCTCTCTACTTTGAACGTTCTGAACCATTTAGAGCTTCTCATCTGGTGCTTTG|ATTCAGCTGATT

ATGCTTTTGC
```
